# Indications and diagnostic yield of small-bowel capsule endoscopy in a real-world setting

**DOI:** 10.1186/s12876-020-01326-8

**Published:** 2020-06-08

**Authors:** André Artan Kharazmi, Saeid Aslani, Malene Fey Kristiansen, Eva Efsen Dahl, Mark Berner-Hansen

**Affiliations:** Digestive Disease Center, Bispebjerg Hospital, University of Copenhagen, Bispebjerg Bakke 23, DK-2400 Copenhagen NV, Denmark

**Keywords:** Diagnostic yield, Small-bowel capsule endoscopy, Real-world, Indications, Obscure gastrointestinal bleeding, Crohn’s disease

## Abstract

**Background:**

Indications and diagnostic yield of small-bowel video capsule endoscopy (SB-VCE) are communicated in recent clinical academic guidelines. However, guidelines are based mainly on relatively few, small, selection-biased studies at experienced centers, and thus we lack information on indications and diagnostic yield of SB-VCE in the real-world community setting. The aim of the study was to evaluate indications and diagnostic yield of SB-VCE in the real-world community setting.

**Methods:**

Our local VCE clinical database was used to identify patients undergoing SB-VCE procedures over a 7-year period (2011–2018). Patients were broadly referred and underwent SB-VCE using PillCam™ SB 2 and SB 3 capsule systems. Procedures were reviewed by local endoscopists, who had undergone similar formal SB-VCE review training. Medical reports of the procedures were composed as such. We retrospectively reviewed all reports and gathered data regarding indications and findings. Diagnostic yield was considered positive if SB-VCE visualized any type of clinically significant pathological finding.

**Results:**

536 SB-VCE procedures in 516 patients were included in final assessment. Patient mean (± SD) age was 50 ± 20 years with approximately even female/male ratio (275:241). The overall proportion of positive findings was 42% (225/536). The two main indications were obscure gastrointestinal bleeding (occult/anemia or overt/active, OGIB) of 46% (246/536) and definite/suspected Crohn’s disease (CD) of 39% (210/536). Positive SB-VCE findings were obtained in 44% (108/246) of procedures with indication of OGIB and in 50% (104/210) of procedures with indication of CD.

**Conclusions:**

The indications for SB-VCE are largely consistent with guidelines but with an apparently relatively low diagnostic yield in our real-world community setting.

## Background

Small-bowel video capsule endoscopy (SB-VCE) has been used as diagnostic tool for a variety of different indications. The importance of clear instructions for optimal clinical use and expectations of SB-VCE has been outlined together with appropriate indications and diagnostic yield in two recent consensus-based guidelines [1, 2].

Guidelines state recommendations about the use of SB-VCE for a few absolute indications: obscure gastrointestinal bleeding (OGIB), known or suspected Crohn’s disease (CD) and small-bowel tumors. Recommendations are mainly based on meta-analyses, systematic reviews and studies conducted at single centers. Some of these studies focus on single indication for SB-VCE with respect to diagnostic yield. Other studies focus on comparison of diagnostic yield of SB-VCE versus other examination techniques such as fiber endoscopic modalities and imaging evaluation of the small-bowel. However, the majority of studies are conducted at experienced centers and with a relatively low number of patients and procedures. As such, they contain the inherent risks of not fully reflecting the real-world community setting [3–8]. This is acknowledged in guidelines as the level of evidence for recommendations are graded and did not reach consistent levels of high evidence, mainly due to risk of bias, indirectness and imprecision [2].

The purpose of the present retrospective observational study was to assess indications and performance expressed in diagnostic yield of SB-VCE in our real-world community setting, using a large unselected number of SB-VCE procedures in an open referral system. The assessment was expected to give an insight into whether real-world community indications and diagnostic yield are in line with clinical guidelines. Accordingly, we believe that the outcomes of this study largely reflect indications for and diagnostic yield of SB-VCE in the real-world community setting.

## Methods

### Patients

All patients were referred to SB-VCE examination at our institution (primary and secondary referral center for OGIB and inflammatory bowel disease) following conventional workup including both upper and lower fiber endoscopic examinations without an established diagnosis. Patients could be referred by physicians practicing in any type of health care center (primary, secondary or tertiary). Yet, the majority of patients were referred by gastroenterologists and surgeons practicing at our institution. Patients being controlled and monitored for chronic disease activity (predominately definite CD) were also referred to SB-VCE examination.

During a 7-year period (2011–2018), 602 SB-VCE procedures were performed for a variety of indications. The SB-VCE procedures were reviewed by 11 local endoscopists that had undergone the same formal SB-VCE review training. SB-VCE reports were collected consecutively in our local database.

SB-VCE reports included the following key parameters for SB-VCE procedure: indication, length, description of bowel visualization, findings and conclusions. Bowel visualization was characterized as being either good or reduced, depending on whether the mucosa was visible for evaluation. If the endoscopist encountered limited visualization and no pathology, the procedure was considered inconclusive and excluded for subsequent analysis. The timing of SB-VCE in the courses of examination for the indications/onset of symptoms varied on a patient case-by-case basis.

Data for this study was extracted manually and retrospectively from 536 SB-VCE reports, and pre-SB-VCE data consisted of patient age, gender and date of procedure. No post procedure data were included.

Due to the retrospective study design and collection of data, some indications were more frequent than others. As such, the diagnostic yield of the two major absolute indications OGIB and CD were chosen to be addressed in more detail.

### Equipment

Medtronic PillCam™ SB2 and SB3 capsule systems were used. The SB2 capsule operating at a fixed picture frequency of 2 frames per second (FPS), while the SB3 capsule operating at a picture frequency of 2 or 6 FPS depending on capsule speed through the gastrointestinal (GI) tract. There was no indication for use of SB-VCE patency capsule in advance of SB-VCE procedures, as MRE scans were performed in selected patients presenting with symptoms, signs or history suggesting mechanical passage issues. The capsule transmitted the acquired images to the data recorder unit (Pillcam™ DR2 and DR3) located outside of the patient’s body. Images were then transmitted from data recorder to workstation (computer). SB-VCE examinations were viewed using the Pillcam™ reader software [9].

### Procedure

SB-VCE procedures were all performed following a standardized protocol. Bowel preparation was initiated the day prior to procedure. Patients were instructed to stay on a clear liquid diet and a bowel lavage was given the evening before and the morning of the procedure. Patients swallowed the capsule and were permitted to go home with a data recorder contained inside a belt. Normal diet was resumed 4 h after capsule ingestion and patients returned with the data recorder the following day.

### Interpretation of results

Indications for SB-VCE were categorized in groups in relation to the general underlying issue. OGIB was defined as occult or overt GI bleeding. Anemia was considered a reliable indication for occult OGIB since occult bleeding would intuitively be the only reason for SB-VCE examination of anemia. CD was categorized as patients either having definite or suspected CD. Defecation disorders consisted of patients suffering from symptoms or signs of predominately diarrhea, alternatively alternating defecation pattern or mucus in stool. Furthermore, indications that were vague or not frequent enough for categorization into respective groups were assembled into a common group named miscellaneous.

Findings were in some instances categorized primarily if they included non-specific findings, such as mucosal changes (erythema and edema) or mucosal lesions (ulcerations, ulcers and erosions).

The definition of clinically significant findings was defined as any type of pathological finding and not necessarily having a direct relation to the indication. These findings were classified as being contributive to diagnostic yield (i.e. positive findings).

As a measurement of consistency and quality control of SB-VCE examinations, the median small-bowel transit time (SBTT) was calculated and compared with historical data reported from healthy individuals.

## Results

### Procedures and patients

**Fig. 1:**
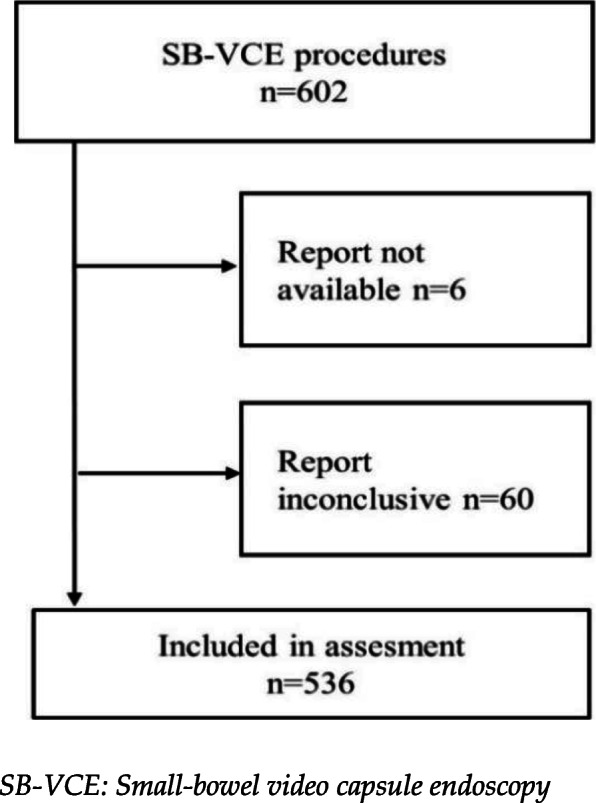
Flowchart. Number of SB-VCE procedures (year 2011–2018) included in assessment

Out of the 602 SB-VCE reports, a total of 66 reports were either not available (6 reports) or evaluated as inconclusive (60 reports) due to external factors (insufficient bowel preparation, small-bowel visualization, inadequate capsule battery time, defects in picture transmission, patient being unable to swallow capsule). Specific causes for inconclusive reports are presented in Table 1. As such, 11% (66/602) of SB-VCE procedures were inadequate for analysis and interpretation leaving 89% (536/602) of procedures, performed in 516 patients, to be included in the final assessment and analysis (see Fig. 1).

**Table 1:** Specific causes for inconclusive small-bowel video capsule endoscopy procedures

Causes for inconclusive procedures	Proportion of inconclusive procedures
Reduced small bowel visualization and/or insufficient small bowel preparation	41% (27/66)
Inadequate capsule battery time	27% (18/66)
Error in picture transmission	11% (7/66)
Patient being unable to swallow capsule	6% (4/66)
Other technical issues	6% (4/66)
No available report	9% (6/66)

Patients were referred across all age groups with increased frequency of patients in their twenties and sixties. Patient mean age was 50 ± 20 (SD, range 17 to 91 years) with approximately even female/male ratio (275/241). A majority of procedures (94%, 506/536) had good visualization of the small bowel, while a minority of procedures (6%, 30/536) were described as having reduced visualization albeit sufficient for diagnostic intend. SBTT was available for a majority of procedures (92%, 492/536). Overall median SBTT was estimated to 4 h and 18 mins (range 1 h to 14 h and 37 mins). No complications in relation to the procedures was registered and no capsule retention was reported.

### Indications

**Fig. 2:**
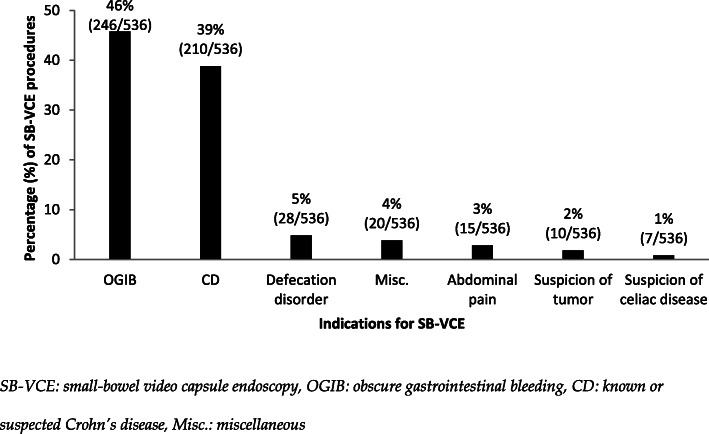
Figure depicting indications for SB-VCE with distribution of total of 536 procedures. Examples of patient indications in the Misc. group: small bowel intussusception, venous thrombosis in the splanchnic circulation, unknown indication and weight loss

The major indications for SB-VCE were OGIB 46% (246/536) and CD 39% (210/536). Only a minority of CD patients, 15% (31/210), were diagnosed with CD before undergoing SB-VCE. Defecation disorder 5% (28/536), miscellaneous 4% (20/536), abdominal pain 3% (15/536), suspicion of tumor 2% (10/536) and suspicion of celiac disease 1% (7/536) were minor indications (see Fig. 2).

### Diagnostic yield

**Fig. 3:**
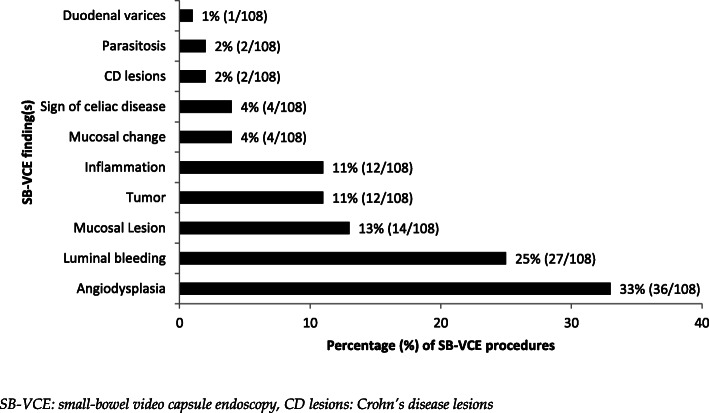
Figure depicting positive findings visualized in the 44% (108/246) of procedures with indication of OGIB: obscure gastrointestinal bleeding

**Fig. 4:**
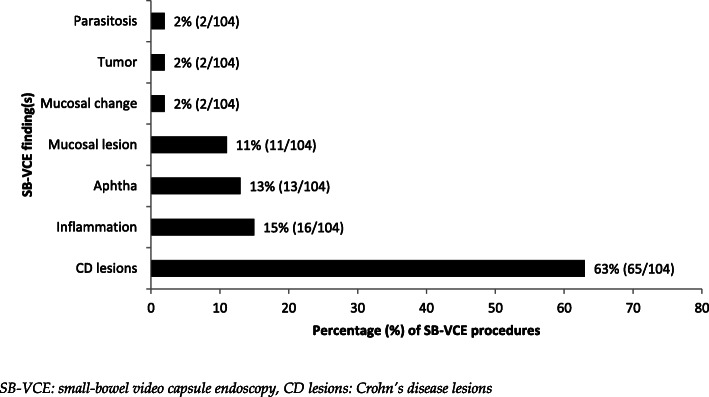
Figure depicting positive findings visualized in the 50% (104/210) of procedures with indication of CD: known or suspected Crohn’s disease

**Fig. 5:**
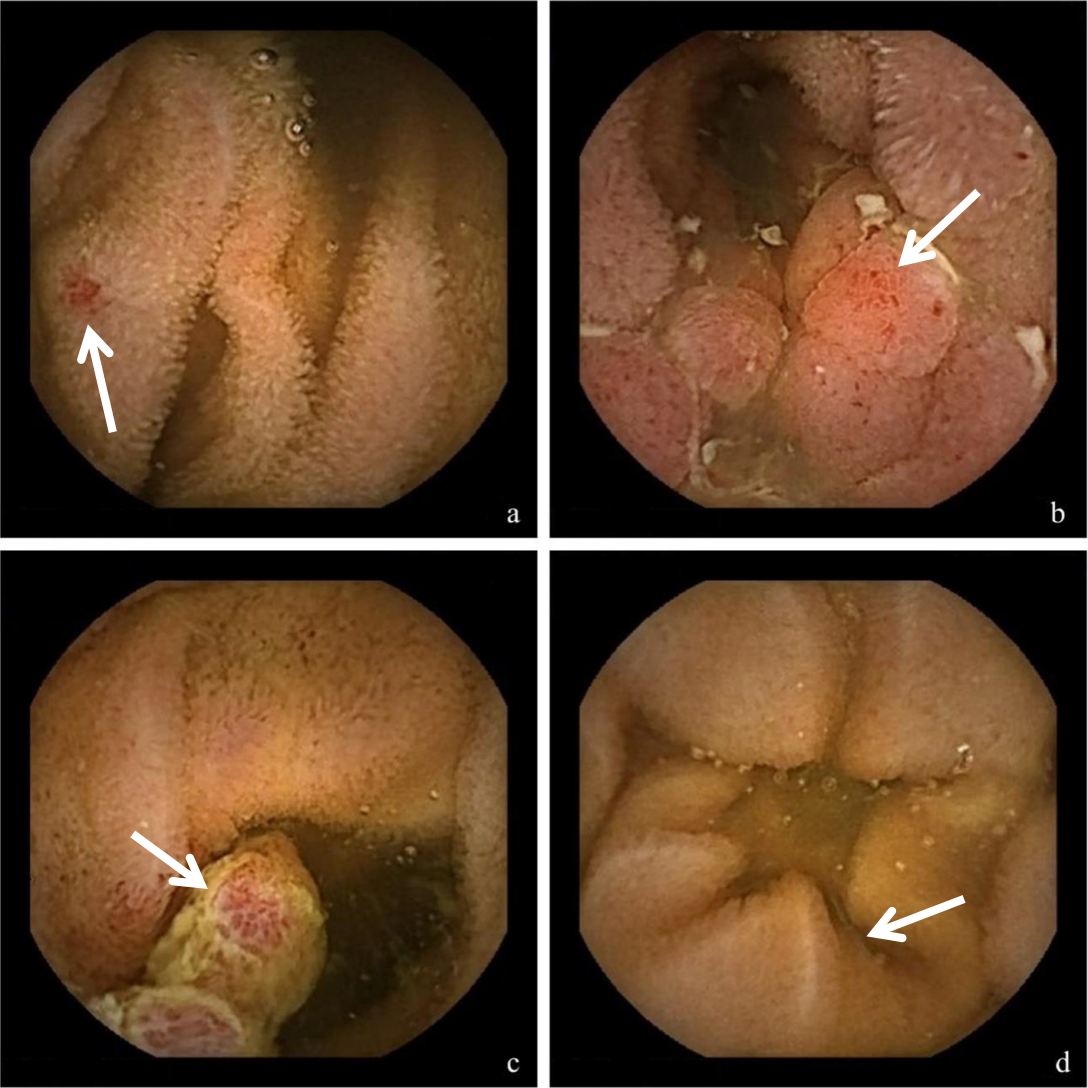
Examples of findings visualized by SB-VCE. **a** Angiodysplasia in patient with OGIB. **b** Nodular inflammation with ulceration in patient with severe Crohn’s disease of the small bowel. **c** Multilobular polyp with ulceration in patient with OGIB. **d** Patchy villous atrophy in patient with OGIB (later duodenal biopsies confirmed the diagnosis of celiac disease)

The overall proportion of positive findings (diagnostic yield) for SB-VCE examinations was 42% (225/536). For the two main indications, OGIB and CD, the proportion of positive findings was 44% (108/246) and 50% (104/210), respectively. Specific findings for main indications OGIB and CD are depicted in Figs. 3, 4 and 5. The most common findings for OGIB indication included angiodysplasia (33%, 36/108), luminal bleeding (25%, 27/108) and mucosal lesion (13%, 14/108). Most common findings for CD indication included CD lesions (63%, 65/104), inflammation (15%, 16/104) and aphtha (13%, 13/104). Some procedures resulted in more than one positive finding during SB-VCE examination.

## Discussion

The objective of this retrospective study was to assess indications and diagnostic yield of SB-VCE in a real-world community setting. We believe that the performance of SB-VCE observed in the present study reflects the real-world community setting in our health system in Denmark and maybe also in other similar health systems, since the assessment was based on a broad non-selective referral system and large number of SB-VCE procedures, outnumbering subjacent studies in recent guidelines [1, 2].

The main indications were OGIB and CD, which is in line with guidelines [1, 2]. However, some deviations were noted when considering some of the infrequent indications (e.g. defecation disorders and abdominal pain). Since SB-VCE was conducted in patients without an established diagnosis after conventional work up, it is possible that a small number of examinations were done with the intent to exclude possible pathology rather than being the appropriate diagnostic tool to obtain a definite and specific diagnosis. This could be valid in relatively few cases, where the indications were ambiguous or questionable.

Furthermore, patients were not always recommended to undergo SB-VCE examination according to a distinct protocol, which is a consequence of the open referral system we have at our institution. This could explain some of the unconventional and uncommon relative indications provided by the referring physician. Finally, the studies referred to in the guidelines are characterized by generally including SB-VCE procedures with distinct indications and conducted at experienced centers [1, 2]. Thus, this might not reflect equivalently the real-world community setting as described in the present study.

The overall diagnostic yield observed in the present study was 42%, which is in line with the estimate of 48% observed in a recent Italian prospective multicenter study by Soncini et al. [10] Dissecting numbers, the diagnostic yield for CD and OGIB indications were apparently lower in the present study as compared to guidelines. According to guidelines [1, 2], the diagnostic yield is expected to be in the range of 47–71% for patients examined for suspected or definite CD. We observed a diagnostic yield of 50% for this indication. As such, the observed 50% diagnostic yield in the present study is in the lower range. Similarly, the observed diagnostic yield of 44% for OGIB indication in the present study was also in the lower range of the reported range of the 30–73% reported in guidelines [1, 2].

We need to acknowledge that comparing indications, and especially diagnostic yield, carries considerable risks of reaching inappropriate conclusions due to differences in study designs, including definitions and other confounders. The definition of what contributes to diagnostic yield is identical to that which is considered of clinical significance, and varies among studies. Thus, as stated in Enns et al. [2], standardized criteria for documenting SB-VCE findings should be developed. This study’s definition was broad and consisted of findings that had any type of pathological association. Based on this definition, the diagnostic yield estimated in present study might be overestimated. Diagnostic yield is generally defined as the likelihood that a test or procedure will provide the information needed to establish a diagnosis. As no post procedure data was incorporated in the study, it was necessary to keep the definition of clinically significant findings broad. Thus, the observed apparent differences in prevalence of positive findings could be due to differences in diagnostic yield concepts.

The variation in the distinction between significant and non-significant finding is also associated with the inherent intra- and interobserver bias of the present study. Having 11 different endoscopists review procedures could be a potential source of interobserver bias. On the other hand, this also reflects real-world community setting, where several physicians practice at the same institution. In a few instances, doubtful findings were conferred with colleagues (i.e. second opinion). Yet, most of the evaluations were concluded by a single reviewer and, in our opinion, likely reflect real-world practice.

The evaluation of the SB-VCE procedures in the present study had a limited degree of standardization and nomenclature, which could contribute to increased interobserver bias, as outlined in other studies [11, 12]. The impact of interobserver variability could not be assessed in this study as each SB-VCE was reviewed by only one endoscopist by default. As such, this could be a potential confounder for diagnostic yield assessment.

A weakness of the present study was the retrospective design and the inherent confounders and bias. Accordingly, SB-VCE procedure data were not uniformly registered. Furthermore, 11% of procedures had to be excluded, as they were not fit-for-purpose due to mainly impaired visualization of mucosa. In addition, given the different level of experience of the endoscopists, a procedure could generate different conclusions (interobserver variability) and thereby affect study findings. However, as SBTT (a consistency and quality control measure) was in the anticipated range [13] and the majority of procedures included in assessment had good visualization, we believe the present study’s findings and conclusions reflect real-world practice by and large.

One could argue that another potential weakness of the present study was the use of both SB2 and SB3 VCE systems, as this could be accompanied by differences in diagnostic yield depending on the system used. However, a recent study comparing these two systems did not support this claim, as only minor differences in diagnostic yield were recorded between the two capsule systems. A more detailed description of mucosal villi alterations is the main advantage of using the newer SB3 system [14].

Finally, the time from the onset of symptoms to SB-VCE examination has been shown to influence the diagnostic yield in patients with indication of OGIB [15, 16]. In concordance with these studies, a recent randomized controlled trial showed that early SB-VCE examination in patients with non-hematemesis GI bleeding could improve bleeding source localization compared to standard care [17]. Accordingly, timing is of importance for diagnostic yield and a limitation of the present real-world study.

## Conclusions

We conclude that indications for SB-VCE in our real-world community setting are largely consistent with guidelines, but with an apparently relatively low diagnostic yield. It is evident that the VCE field needs further attention for standardization and refinement of definitions. Finally, adoption of a standardized referral protocol could also increase SB-VCE outcomes.

## Data Availability

The datasets used and analysed in the current study are available from the corresponding author upon reasonable request.

## Abbreviations

SB-VCE: small-bowel video capsule endoscopy
OGIB: obscure gastrointestinal bleeding
CD: known or suspected Crohn’s disease
GI: gastrointestinal
SBTT: small-bowel transit time
CD lesions: Crohn’s disease lesions
